# A Self‐Amplifying RNA Lipid Nanoparticle (saRNA‐LNP) Vaccine Provides Effective Protection Against Porcine Epidemic Diarrhea

**DOI:** 10.1155/tbed/3115893

**Published:** 2026-05-06

**Authors:** Jinqi Shu, Han Zhang, Fang Wang, Hanyu Zhang, Xin Liu, Keshu Liu, Yinhe Zha, Wenqin Chai, Jianghui Yao, Yujun Lin, Chao Wu, Xiaomin Zhao

**Affiliations:** ^1^ College of Veterinary Medicine, Northwest A&F University, Yangling, 712100, Shaanxi, China, nwsuaf.edu.cn; ^2^ Engineering Research Center of Efficient New Vaccines for Animals, Ministry of Education, Yangling, 712100, Shaanxi, China, moe.edu.cn; ^3^ Hangzhou ISEEVAX Pharmaceutical Technology Co., Ltd., Hangzhou, 310018, Zhejiang, China

**Keywords:** lipid nanoparticles, porcine epidemic diarrhea virus, self-amplifying RNA vaccine

## Abstract

The development of vaccine platforms capable of inducing sustained, potent, and cost‐effective immunity remains a major challenge in veterinary vaccinology, particularly for coronaviruses, which are often associated with short‐lived vaccine‐induced protection. Here, we establish a self‐amplifying RNA (saRNA) lipid nanoparticle (LNP) vaccine platform and evaluate its application using porcine epidemic diarrhea virus (PEDV) as a representative coronavirus model. A single vaccination elicited robust specific humoral and cellular immune responses. Vaccinated piglets exhibited significantly higher specific IgG and neutralizing antibody titers of PEDV‐S (1:160) at 28 days postvaccination (dpv). Furthermore, elevated levels of serum IFN‐γ and IL‐4, along with enhanced CD4^+^ and CD8^+^ T cells, were observed. Vaccinated pregnant sows exhibited significantly higher PEDV neutralizing antibody titers (~1:224 or 256) in serum and colostrum samples. The saRNA‐LNP vaccine conferred both active immune protection against PED in immunized piglets and passive immunity in neonates via colostrum‐derived antibodies from vaccinated sows. Notably, the truncated spike protein antigen outperformed the full‐length spike protein in terms of immunogenicity, revealing the importance of careful testing in designing this PEDV saRNA vaccine. The saRNA‐LNP system represents a dose‐sparing, single‐dose vaccine platform with the potential to extend protective immunity, enhance maternal antibody responses, and reduce vaccination costs for PEDV and other veterinary coronaviruses.

## 1. Introduction

The self‐amplifying RNA (saRNA) vaccines represent a distinct class of RNA vaccines in which the antigen‐encoding sequence is embedded within an alphavirus‐derived replicon, most commonly originating from Venezuelan equine encephalitis virus (VEEV), Semliki Forest virus (SFV), or Sindbis virus (SINV) [1–3]. In contrast to conventional nonreplicating mRNA vaccines, saRNA vaccines encode viral nonstructural proteins (nsP) 1–4 that form an RNA‐dependent RNA polymerase complex, enabling intracellular RNA amplification and prolonged antigen expression from a substantially lower input dose [3, 4].

Among these platforms, VEEV‐based replicons are the most widely used due to their high replication efficiency and broad cell tropism [5]. Multiple preclinical studies have demonstrated that VEEV‐derived saRNA vaccines formulated in lipid nanoparticles (LNPs) can induce robust humoral immune responses at doses 10‐ to 100‐fold lower than those required for nonreplicating mRNA vaccines [6–8]. For example, VEEV saRNA vaccines encoding viral surface antigens have been reported to induce neutralizing antibody titers in the range of 10^3^–10^5^ (ID_50_) in mice and nonhuman primates, with seroconversion rates exceeding 90% after a single immunization [7]. In comparison, nonreplicating mRNA vaccines typically require higher doses and booster immunizations to achieve comparable antibody levels [6, 8].

However, despite these advantages, existing saRNA platforms also exhibit notable limitations. Excessive innate immune activation driven by replicase expression can suppress antigen translation, while suboptimal replicon design may result in unstable RNA replication or truncated antigen expression [9, 10]. To address these issues, recent efforts have focused on optimizing VEEV replicon elements, including modifications to the nsP coding region, subgenomic promoter strength, and untranslated regions (UTRs), to balance RNA amplification with sustained antigen production [11, 12]. Nevertheless, most of these optimizations have been evaluated in small‐animal models, and their translational relevance in large natural hosts remains insufficiently characterized [13, 14]. Moreover, recent advances in optimizing the replicon design, including codon optimization and modifications to UTRs, have demonstrated promising results in improving antigen expression and immune response durability [4, 12].

Porcine epidemic diarrhea virus (PEDV) is an enteric coronavirus causing severe diarrhea and high mortality in neonatal piglets, making it one of the most economically devastating swine diseases worldwide [15, 16]. Similar to other coronaviruses, PEDV vaccines—whether inactivated, live‐attenuated, or mRNA‐based—are generally associated with short‐lived protective immunity, typically lasting 3–6 months, and require frequent revaccination [17–19]. Reported neutralizing antibody titers induced by conventional PEDV vaccines are often modest, with variable seroconversion rates and incomplete protection against heterologous strains [20]. Importantly, protection of neonatal piglets relies predominantly on maternal lactogenic immunity, which remains a major challenge for current vaccine strategies [21, 22].

These characteristics position PEDV as a stringent and clinically relevant model for evaluating whether saRNA vaccine platforms can overcome the limitations of existing coronavirus vaccines. We hypothesized that an optimized VEEV‐based saRNA‐LNP platform, designed to enhance antigen expression kinetics while minimizing replicase‐associated translation inhibition, could induce higher neutralizing antibody levels, extend the duration of protective immunity, and improve maternal antibody transfer. Therefore, in this study, we established and validated an optimized saRNA‐LNP vaccine platform, using PEDV as a proof‐of‐concept coronavirus antigen, and systematically evaluated its immunogenicity, protective efficacy, and durability in the natural host.

## 2. Materials and Methods

### 2.1. Design, Cloning, and Production of mRNA and saRNA Expression Plasmids

The recombinant plasmids pUC57‐saRNA‐0 and pUC57‐saRNA‐1 contain the replicon of the VEEV (GenBank: L01443.1) as the backbone that includes 5^′^Cap, 5^′^UTR, nonstructural polyprotein (nsP1‐4), subgenomic promoter, and 3^′^UTR, followed by either a 30 nt or a 120 nt poly(A) tail. The T7 promoter sequence was placed upstream of the 5^′^Cap, and the firefly luciferase (FLuc) reporter gene was cloned between the promoter and 3^′^UTR. The pUC57‐saRNA‐2 construct derived from pUC57‐saRNA‐1 with targeted mutation of nsP2. For vaccine development, the S glycoprotein from the PEDV–WH strain served as the target antigen. The sequences encoding the S1 subunit (including the N‐terminal signal residues 1–24) and a partial S2 subunit (residues 735–785) were cloned into the optimized pUC57‐saRNA‐2 construct in replacement of the reporter gene. All plasmid sequences were verified by Sanger sequencing. Recombinant plasmids were purified using EndoFree Plasmid Maxi Kits (QIAGEN, Germany) and linearized with *AsiS*I restriction enzyme (NEB, USA).

### 2.2. Production of mRNA and LNP Encapsulation

The saRNA and nonreplicating mRNA were synthesized via in vitro transcription using T7 RNA polymerase with linearized plasmid DNA as a template. The transcription reaction consisted of linearized template DNA, N1‐methylpseudouridine‐5^*′*^‐triphosphate (Novoprotein, China), CAP GAU m7G(5^*′*^)ppp(5")(2^*′*^0MeA)pU (SYNTHGENE, China), and components from the T7 High‐Yield RNA Transcription kit (Novoprotein, China). Reactions were incubated at 37°C for 2.5 h, followed by purification using magnetic beads (Vazyme, China).

For LNP encapsulation, purified RNA was processed using a microfluidic synthesizer (Micro Nano, China). The oil phase (lipid mixture) was diluted 1:1 in anhydrous ethanol before loading. According to N/P = 5:1, the volume ratio of the oil phase to the water phase is 1:3. The RNA sample was diluted according to the calculations to the required concentration using citrate buffer at pH 4.5. Encapsulation was conducted at room temperature. The aqueous phase (RNA solution) and oil phase were injected into the microfluidic chip at a flow rate ratio of 3:1 (total flow rate of 12 mL/min), as controlled by the instrument software. The resulting LNP formulation was diluted in 10× volume of sucrose buffer and then concentrated ~60‐fold by centrifugation (2000 × *g*). Finally, samples were sterile‐filtered and analyzed for RNA concentration and encapsulation efficiency.

### 2.3. Fluorescence Imaging in Mouse

Female C57BL/6 mice (6–8 weeks old) were purchased from Shanghai SLAC Laboratory Animal Co., Ltd. During in vivo optical imaging, mice were anesthetized with 2% isoflurane delivered via a gas anesthesia system. Euthanasia was performed by cervical dislocation upon experiment completion. All procedures conducted by protocols approved by Institutional Animal Care and Use Committee (IACUC) of Hangzhou ISEEVAX Pharmaceutical Technology Co., Ltd (Approval Number. ISEEVAX‐MO‐2024‐015). Strict measures were implemented to ensure animal welfare, including the minimization of animal numbers.

For the imaging study, C57BL/6 mice were intramuscularly injected with 5 μg of LNP‐formulated FLuc‐tagged mRNA or saRNA. At predetermined time points (1, 3, 8, 15, 22, and 30 days postinjection), mice were anesthetized with 2% isoflurane and subsequently administered 3 mg D‐luciferin via intraperitoneal injection. Bioluminescence imaging was performed 10 min postinjection using an IVIS Spectrum Imaging System (PerkinElmer, USA), with quantitative data expressed as total flux (in photons per second per square centimeter per steradian).

### 2.4. Vaccination and Challenge Experiments

Pigs were sourced from Anji Yuanji Animal Farm (Anji County, Zhejiang, China). Euthanasia was administered via intramuscular (IM) injection of Zoletil (tiletamine–zolazepam, 10 mg/kg) following experimental protocols. Experiments with pigs were approved by IACUC of Hangzhou ISEEVAX Pharmaceutical Technology Co., Ltd (Approval Number. ISEEVAX‐SW‐2024‐011), with strict adherence to protocols minimizing animal suffering and sample sizes. Healthy, susceptible piglets (confirmed negative for PEDV, TGEV, RV, PRRSV, and PCV2 by qRT‐PCR) were used throughout.

#### 2.4.1. Comparison of Antibody Responses Between Full‐Length and Truncated saRNA Vaccine Candidates

Nine 9‐day‐old piglets were randomly divided into three groups (*n* = 3/group): full‐length saRNA‐S‐LNP group (25 μg dose/piglet, single IM neck injection), truncated saRNA‐S‐LNP group (25 μg dose/piglet, single IM neck injection), and mock group (LNP mock). All groups were housed separately. Serum samples were collected at 7, 21, and 70 dpv for analysis of antibody responses.

#### 2.4.2. Comparative Immune Responses Between saRNA and Nonreplicating mRNA Vaccines

Twelve 9‐day‐old piglets were randomly divided into four groups (*n* = 3/group): saRNA group (25 μg PEDV‐S saRNA vaccine/piglet, single IM neck injection), mRNA group (25 μg nonreplicating mRNA vaccine/piglet, single IM neck injection), CIV group (commercial inactivated vaccine, two doses as per manufacturer’s protocol), and mock group (LNP mock, single dose). All groups were housed separately. Serum samples were collected at 7, 14, 21, and 28 dpv for immunological assays.

#### 2.4.3. Dose Optimization Study

For dose evaluation, 12 9‐day‐old piglets were assigned to four groups (*n* = 3/group): groups saRNA‐10, saRNA‐20, and saRNA‐50 given 10, 20, and 50 μg saRNA vaccine/piglet, and the mock (LNP) group. Each piglet received a single IM neck injection. Sampling occurred at 7, 14, 21, 28, and 35 dpv.

#### 2.4.4. Immunization Frequency Study

Twelve 9‐day‐old piglets (*n* = 3/group) were allocated: sa‐Once group receiving single 20 μg/piglet saRNA at Day 0; sa‐Twice group given prime‐boost 20 μg/piglet saRNA at Days 0 and 14; CIV group receiving CIV (prime‐boost per protocol); and mock group, LNP mock. Each piglet received a single IM neck injection. Serum samples were collected at 7, 14, 21, and 28 dpv.

#### 2.4.5. Viral Challenge in Actively Immunized Piglets

Ten piglets were divided into saRNA group (*n* = 5, 20 μg saRNA for single IM neck injection) and mock (*n* = 5, LNP). At 21 dpv (selected based on antibody kinetics and age), piglets were orally challenged with PEDV–WH strain (2.0 × 10^6.5^ TCID_50_/piglet). Fecal samples were collected daily to monitor clinical conditions and assess diarrhea severity using standardized scoring (Table 1) [17, 23–25]. Piglets were humanely euthanized and necropsied at 3 dpc (*n* = 1 per group) and 10 dpc (*n* = 4 per group) with intestinal tissue and content collected for analysis.

**Table 1:** Score sheet for clinical conditions of the piglets challenged with porcine epidemic diarrhea virus^a^.

Category items	Status level	Category score	Total score/piglet
Feces	Normal (formed)	0	3
	Soft, paste‐like, or mushy	1	
	Watery	2	
Mental state	Normal	0	
	Somber	1	

^a^Piglets died during the experiment were directly scored as “4.”

#### 2.4.6. Passive Immunization Study

Ten pregnant sows were immunized at 28 days prefarrowing: vaccination (*n* = 5; 50 μg saRNA/pig, IM neck injection) and control (*n* = 5, LNP). Serum (14, 21, and 28 dpv) and colostrum (0–5 days postfarrowing) were analyzed. On the fifth day after birth, newborn piglets from sows in the vaccination group and challenged control group were challenged with the PEDV–WH strain (2.0 × 10^6.5^ TCID_50_/piglet, *n* = 25/group, respectively). The blank control group received no challenge (*n* = 25). Fecal samples were collected daily to monitor clinical conditions and assess diarrhea severity using standardized scoring [17, 23–25]. Piglets were humanely euthanized and necropsied at 3 dpc (*n* = 2), with intestinal tissue and content collected for analysis.

### 2.5. ELISA for Detection of Serum‐Specific Antibodies

Serum IgG and IgA antibodies specific to the PEDV‐S protein were determined by an inhouse developed indirect ELISA assay which includes the 96‐well plates precoated with recombinant PEDV‐S protein (1 µg/mL) as well as verified PEDV‐positive and PEDV‐negative swine sera served as controls, all stored at 4°C. Samples were diluted 100‐fold in dilution buffer and added into the S‐coated wells (100 µL/well). After 1 h incubation at 37°C, the plates were washed and incubated for 30 min with horseradish peroxidase (HRP)‐conjugated goat anti‐pig IgG or IgA (1:5000 dilution). Subsequent washes were performed before enzymatic development using 3,3^′^, 5,5^′^‐tetramethylbenzidine (TMB) as the substrate (15 min, 37°C). Reactions were stopped with 100 µL of 1% sodium dodecyl sulfate, and absorbance was measured at 650 nm using a BioTek microplate reader (USA). All samples were assayed in triplicate wells. S/P values were calculated as (OD_S_ − OD_N_)/(OD_P_ − OD_N_), where OD_S_ represents OD of the serum samples; OD_N_, that of the negative control serum (OD < 0.25); and OD_P_, that of the positive reference serum (OD > 0.5). Criteria for determining specific IgG antibodies: S/P values ≥0.5 were considered positive, and ≤0.4, negative. Criteria for determining specific IgA antibodies: S/P values ≥0.3 were considered positive, and ≤0.199, negative.

### 2.6. Virus Neutralization Test

Serum samples were heat‐inactivated at 56°C for 30 min and serially diluted twofold. Viral stocks were standardized to 200 TCID_50_ in serum‐free DMEM. Equal volumes of virus suspension and diluted serum samples were combined and incubated at 37°C for 1 h. The mixtures (100 μL) were inoculated onto Vero cells in 96‐well plates at about 80% confluence, with inclusion of cell controls (uninfected) and viral controls (0.1 TCID_50_ and 100 TCID_50_). After adsorption for 1.5 h at 37°C, the inocula were aspirated and the infected wells were washed twice with PBS, and maintenance medium (100 μL) containing trypsin (10 μg/mL) was added into the wells [26]. The plates were incubated at 37°C with 5% CO_2_ for 72 h, with daily microscopic assessment for CPE (Figure S1). The readings were valid when the uninfected cell control and viral control with 0.1 TCID_50_ remained CPE‐negative, while the high‐dose control with 100 TCID_50_ consistently exhibited CPE in all wells. Neutralizing antibody titers were calculated from CPE observations using Reed–Müench method [17, 27].

### 2.7. Flow Cytometry

Peripheral blood lymphocytes from piglets were isolated and resuspended in PBS (100 μL). Cells were stained with SPDR antiporcine CD3 (5 μL/sample; SouthernBiotech, USA), rabbit antiporcine CD4 (1 μL/sample; Bioss, China), PE antiporcine CD8 (5 μL/sample; SouthernBiotech, USA). Samples were incubated at 4°C in the dark for 30 min, centrifuged at 300 × *g* for 5 min, and washed twice with PBS. Subsequently, cells were stained with BF488‐conjugated goat anti‐rabbit IgG (1 μL/sample; Bioss, China) and washed again. Finally, cells were resuspended in 300 μL PBS and analyzed using a CytoFLEX flow cytometer (Beckman Coulter, USA). Data were processed using CytExpert or FlowJo software (version 10.8.1).

### 2.8. Detection of Serum Cytokine Level

Serum concentrations of IL‐4 and IFN‐γ in piglets were quantified using commercial ELISA kits (Porcine IL‐4: Solarbio, China; Porcine IFN‐γ: Solarbio, China) following manufacturer protocols. Briefly, diluted serum samples and provided standards were added to precoated plates and incubated at 37°C for 1 h. After five PBST washes, plates were incubated with HRP‐conjugated detection antibodies (37°C, 20 min). Following additional washes, TMB substrate was added, and optical density was measured at 450 nm after 20 min incubation at 37°C.

### 2.9. Clinical and Histopathological Evaluation

Postchallenge, disease severity in piglets was assessed through clinical observations, including condition scores and fecal consistency. At necropsy, jejunal tissues were harvested, fixed in 4% neutral‐buffered formalin, and processed for histopathological analysis. Tissues were dehydrated, cleared in xylene, paraffin‐embedded, sectioned (4–5 μm), and mounted on slides. Tissue sections were subjected to hematoxylin and eosin (H&E) staining and immunohistochemical (IHC) analysis. For IHC detection, slides were incubated with a laboratory‐generated monoclonal antibody specific for the PEDV‐S protein, followed by HRP‐conjugated goat anti‐mouse IgG (H + L) secondary antibody (Beyotime, China), enabling visualization of viral antigens.

### 2.10. Quantification of PEDV RNA by qRT‐PCR

To assess PEDV RNA expression kinetics posttransfection with RNA‐LNP vaccine formulations, 293T cells seeded in 6‐well plates were transfected with 2.5 µg/well of mRNA‐LNP and harvested at designated time points (0, 6, 12, 24, 48, and 72 h posttransfection). Cells were washed with PBS, lysed using Western/IP lysis buffer (Beyotime, China), and subjected to RNA extraction. Complementary DNA (cDNA) was synthesized using HiScript II Reverse Transcriptase (Vazyme Biotech, China). qRT‐PCR was performed with Taq Pro HS Universal Probe Master Mix (Vazyme Biotech, China) under standardized cycling conditions. The *PEDV-S* gene was amplified using the following primer/probe set: forward primer (5^′^‐TACCAGCTGT ACCTGCACAA‐3^′^), reverse primer (5^′^‐TGTGCTCACTCATGTAGGCA‐3^′^), and probe (5^′^‐CCAATGCCACCGCCCGGCTG‐3^′^). A series of 10‐fold dilutions of mRNA‐LNP, equivalent to 1 × 10^−7^ to 1 ng per reaction mixture, were prepared to generate calibration curves and were run in parallel with the test samples.

Viral RNA copy numbers in fecal swabs were quantified using TaqMan‐based qRT‐PCR. Viral RNA was extracted from centrifuged (10,000 × *g*, 10 min) 10% fecal swab supernatants and reverse‐transcribed. The *PEDV* nucleocapsid (*N*) gene was targeted using the following primers and probes: sense, 5^′^‐GTCTGAAAAGCCAATCATTC‐3^′^; antisense, 5^′^‐TTGCCTCTGTTGTTACTC‐3^′^; and probe 5^′^‐CTGTTGTTGCCATTGCCACGA‐3^′^ [17]. The viral RNA content was determined from the calibration curve of the serially 10‐fold diluted recombinant plasmid containing PEDV N coding region.

### 2.11. Western Blot

To assess in vitro protein expression mediated by the RNA‐LNP vaccine, 293T cells seeded in 6‐well plates were transfected with 2.5 µg/well of mRNA‐LNP formulation. Cells were harvested at specified intervals posttransfection (0, 6, 12, 24, 48, and 72 h), washed with PBS, and lysed using Western/IP lysis buffer (Beyotime, China). Lysates were mixed with 5× SDS–PAGE loading buffer (Beyotime, China), resolved by SDS–PAGE, and transferred onto nitrocellulose membranes (Pall Corporation, USA). Membranes were blocked with 5% nonfat milk, probed with a lab‐generated anti‐PEDV‐S polyclonal antibody (1:1000 dilution), and incubated with HRP‐conjugated sheep anti‐pig IgG secondary antibody (1:1000 dilution, Solarbio, China). Protein bands were visualized using enhanced chemiluminescence (ECL), and densitometric analysis was conducted to compare S protein levels relative to β‐tubulin.

### 2.12. Statistical Analysis

Data are presented as mean ± SD. Statistical analyses were performed using GraphPad Prism 8 (GraphPad Software, USA). Differences between experimental groups were assessed using mixed‐effects analysis or one‐way ANOVA, followed by Tukey’s multiple comparisons test. Significance levels are denoted as follows: *p*  < 0.05 ( ^∗^), *p*  < 0.01 ( ^∗∗^), and *p*  < 0.001 ( ^∗∗∗^).

## 3. Results

### 3.1. Platform Optimization of the saRNA Vaccine Constructs

**Figure 1:**
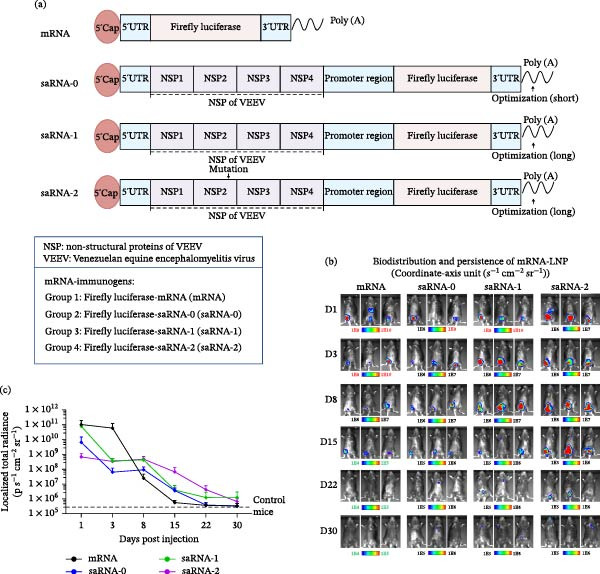
Platform optimization of the saRNA vaccine constructs. (a) Schematic representation of mRNA and saRNA constructs. (b, c) Biodistribution and persistence of RNA‐LNP. Mice were intramuscularly injected with luciferase‐labeled RNA‐LNP, anesthetized at different days postinjection, and D‐luciferin intraperitoneally administered for bioluminescence imaging (b) and quantification of fluorescence intensity as mean ± SD of three mice at each time point (c).

Nonstructural protein 2 (nsP2) acts as the replicative core of saRNA. Its enzymatic activity and immunogenicity could be optimized to enhance the replication efficiency while mitigating cytotoxicity [3, 11, 28]. The poly(A) tail acts as a key regulator of translation and stability, and rational design of its length and sequence can enhance mRNA formation, improve translation efficiency, and prolong mRNA half‐life [29–31]. To enhance target protein expression and improve its immunogenicity and durability, we optimized the nsP2 and poly(A) tails of saRNA constructs, generating three distinct variants, and a nonreplicating mRNA construct was also designed for comparison (Figure 1a). To evaluate mRNA persistence in vivo, four FLuc‐labeled mRNA formulations encapsulated in LNPs were intramuscularly administered to C57BL/6 mice. Bioluminescent imaging revealed detectable luciferase activity across all groups (Figure 1b,c). On Day 1, the mRNA and saRNA‐1 groups exhibited the highest luminescence intensity (one to two orders of magnitude greater than other groups). However, luminescence in the mRNA group sharply declined from Days 3 to 8 (~1000‐fold reduction), whereas the saRNA groups (saRNA‐0, saRNA‐1, and saRNA‐2) maintained stable expression during this period, with saRNA‐1/saRNA‐2 showing significantly higher signals than saRNA‐0. Longitudinal tracking demonstrated distinct persistence profiles: The mRNA group neared baseline signal by Day 15; the saRNA‐0 group by Day 22; and the saRNA‐1/saRNA‐2 groups remained detectable until Day 22 but diminished by Day 30. Notably, the saRNA‐2 construct achieved superior sustained expression, with both higher fluorescence intensity and prolonged duration relative to other variants.

### 3.2. Characterization of a PEDV‐S saRNA Vaccine

**Figure 2:**
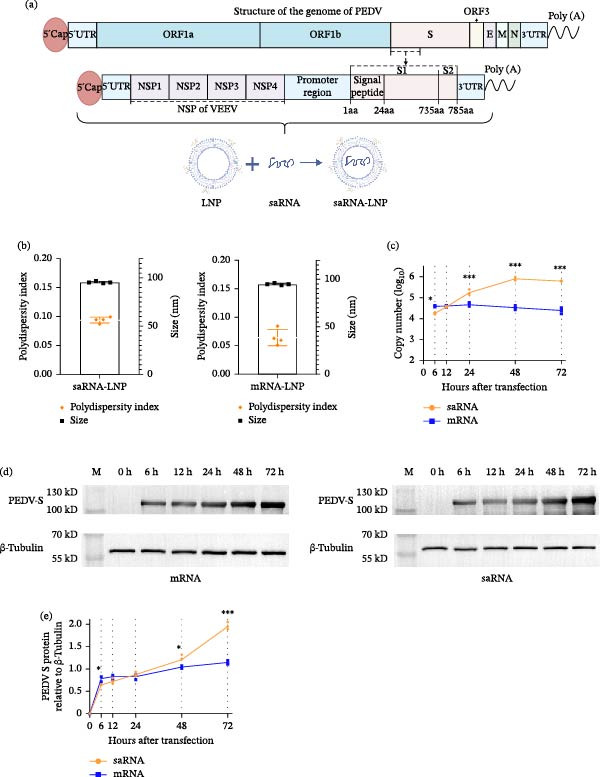
Production and characteristics of PEDV‐S mRNA and saRNA vaccines. (a) PEDV‐S saRNA vaccine design schematic. (b) Average particle size and PDI of RNA‐LNPs by dynamic light scattering. (c) qRT‐PCR quantification of the transcription efficiency in 293T cells transfected with saRNA‐ or mRNA‐LNPs at indicated time points. (d) Western blotting analysis PEDV‐S protein expression in 293T cells transfected with saRNA‐ or mRNA‐LNPs at indicated time points. (e) Densitometric quantification of PEDV‐S expression relative to β‐tubulin based on the blots of (d). Results in panels (c) and (e) were shown as mean ± SD of three independent experiments.

Based on the optimized saRNA construct, we developed a PEDV‐S saRNA vaccine encoding codon‐optimized S1 and a partial S2 domain (residues 735–785 aa) (Figure 2a). A nonreplicating mRNA vaccine with identical antigen sequences was used for comparative evaluation. Both mRNA and saRNA vaccine constructs were encapsulated in LNP. The saRNA‐LNP exhibited an average particle size of 95.5 nm (polydispersity index [PDI] = 0.094) and >96.9% encapsulation efficiency, while nonreplicating mRNA‐LNP measured at 94.9 nm (PDI = 0.064) with >97.1% encapsulation efficiency (Figure 2b). A PDI value lower than 0.1 confirmed that the nanoparticles were uniformly dispersed, and the ~95 nm size optimizes cellular uptake and biodistribution [32, 33]. These characteristics, together with >96% encapsulation efficiency, ensure potent RNA delivery. Quantitative PCR analysis demonstrated that nonreplicating mRNA elicited higher antigen expression levels in the 293T cells than saRNA during the early‐phase posttransfection (<12 h; Figure 2c). However, expression profiles converged at 12 h, after which saRNA‐mediated antigen production surpassed that of nonreplicating mRNA. Western blotting verified robust S protein expression of both platforms (Figures 2d,e), showing a similar temporal pattern: Nonreplicating mRNA initially yielded higher protein quantities, with equivalence achieved by 24 h and subsequent superiority of saRNA expression. These in vitro findings collectively confirm the self‐amplifying nature and enhanced translational capacity of saRNA vaccine technology.

### 3.3. Humoral Immune Responses and Neutralizing Antibodies Against PED by Nonreplicating mRNA and Self‐Amplifying mRNA Vaccines in Piglets

**Figure 3:**
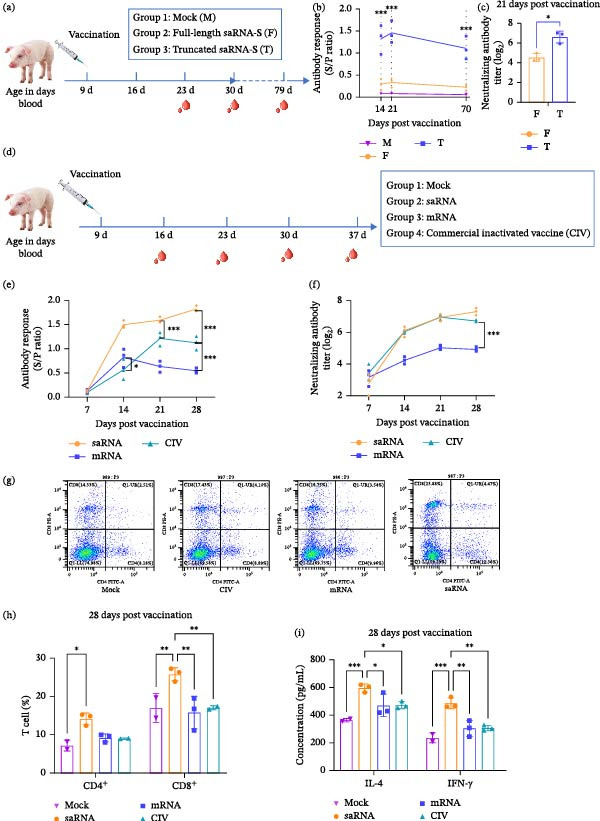
Humoral and cellular immune responses in piglets immunized with self‐amplifying mRNA‐S‐LNP vaccine and saRNA‐S‐LNP vaccine against PEDV. (a) Vaccination schedule for comparison of full‐length saRNA‐S‐LNP (25 μg/pig), truncated saRNA‐S‐LNP (25 μg/pig), or PBS (control) at 9 days postnatal. Serum samples were collected at 14, 21, and 70 dpv for (b) PEDV‐S specific antibody responses by ELISA. (c) Neutralizing antibody titers against PEDV–WH (G2a strain) at 21 dpv. (d) Vaccination schedule: piglets received saRNA (25 μg/piglet), mRNA (25 μg/piglet), commercial inactivated vaccine of PEDV‐S, or LNP mock (M) at 9 days postnatal. Blood samples were collected at 7, 14, 21, and 28 dpv for analysis of PEDV‐S‐specific serum antibodies by indirect ELISA (e) or of neutralizing antibody titers against PEDV–WH (G2a strain) (f). (g) Representative flow cytometry plots (28 dpv) and (h) frequencies of CD3^+^CD4^+^ and CD3^+^CD8^+^ T cells in peripheral blood lymphocytes (gated per 1 × 10^5^ cells). (i) Serum IL‐4 and IFN‐γ concentrations at 28 dpv. Data were presented as mean ± SD of three piglets per group. Significance differences were analyzed using one‐way ANOVA ( ^∗^ *p* < 0.05,  ^∗∗^ *p* < 0.01,  ^∗∗∗^ *p* < 0.001).

We first compared the immunogenecity of two saRNA vaccine candidates encoding either the full‐length S or a truncated S (amino acids 1–785) protein in piglets (Figure 3a). Contrary to expectations, the truncated saRNA‐S elicited higher antibody responses, including increased neutralizing antibody titers, than its full‐length counterpart (Figure 3b,c), suggesting its superior suitability for saRNA‐based vaccine development.

To further evaluate the immunogenicity of the truncated saRNA‐S vaccine candidate in PEDV’s natural host, piglets were divided into four groups: saRNA vaccine, nonreplicating mRNA vaccine, CIV, and LNP mock (M) (Figure 3d). No adverse effects were observed in the piglets postvaccination. Serum samples collected at 7, 14, 21, and 28 days dpv were analyzed for antibodies by indirect ELISA and neutralization assays. The saRNA group exhibited significantly higher specific IgG response at 14 dpv with sustained elevation through 28 dpv that consistently outperformed the mRNA and CIV groups (*p* < 0.001) (Figure 3e). The neutralization assay revealed comparable potency between the groups saRNA and CIV from 7 to 21 dpv. (Figure 3f). In the group saRNA, however, the neutralization antibodies continued to increase till Day 28, as compared to the decline in the group CIV. Of note, the antibody levels in the mRNA group were much lower than saRNA and even with CIV (Figure 3e,f). These results demonstrate that the saRNA vaccine induces robust and sustained antibody responses, surpassing both nonreplicating mRNA and CIV.

### 3.4. Evaluation of Cellular Immune Responses in Piglets

To further characterize the cellular immune responses induced by the truncated saRNA‐S vaccine candidate, blood samples were collected from the piglets at 28 dpv for isolation of peripheral blood lymphocytes and sera (Figure 3d). Flow cytometric analysis revealed significantly elevated percentages of CD8^+^ T cells in the saRNA vaccine group compared to the nonreplicating mRNA, CIV, and mock groups (Figure 3g,h). The group saRNA also exhibited markedly higher serum concentrations of both Th1‐associated IFN‐γ and Th2‐associated IL‐4 than the other groups (Figure 3i). These results demonstrate that the saRNA vaccine‐elicited robust cellular immune responses, significantly outperforming both nonreplicating mRNA and CIV in inducing T‐cell activation and cytokine production.

### 3.5. Optimization of PEDV‐S saRNA Vaccine Immunization Strategy

**Figure 4:**
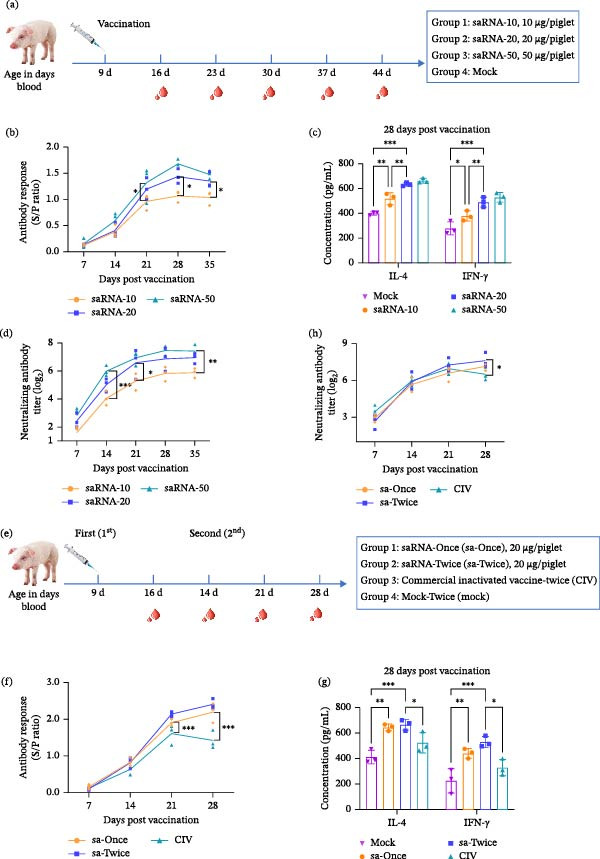
Optimization of PEDV‐S saRNA vaccine immunization schedule. (a) Dose–response study: piglets received 10, 20, or 50 μg saRNA vaccine. (b) PEDV‐S‐specific antibody responses. (c) Serum IL‐4 and IFN‐γ at 28 dpv. (d) Neutralizing antibody titers against PEDV–WH (G2a strain) over time. (e) Immunization frequency study: one verus two doses. (f) PEDV‐S‐specific antibody responses. (g) Cytokine levels at 28 dpv. (h) Neutralizing antibody titers against PEDV–WH (G2a strain) over time. Data were presented as mean ± SD of three piglets per group. Significance differences were analyzed using one‐way ANOVA ( ^∗^ *p* < 0.05,  ^∗∗^ *p* < 0.01,  ^∗∗∗^ *p* < 0.001).

To establish an optimized immunization strategy of platform‐derived PEDV vaccine for piglets, we first evaluated the dose–response relationship. The 9‐day‐old piglets received a single‐neck IM injection of the saRNA vaccine at doses of 10, 20, or 50 μg/head, with LNP as mock (Figure 4a). Serum analysis revealed similar antibody response kinetics across all vaccine groups, peaking at 28 dpv with the 10 μg group showing lower S/P values than the other two groups (Figure 4b). Notably, no significant difference in antibody response was observed between the 20 μg (saRNA‐20) and 50 μg (saRNA‐50) groups through 35 dpv. Cellular immune responses were assessed at 28 dpv via ELISA quantification of IL‐4 and IFN‐γ. Higher IL‐4 and IFN‐γ levels were observed dose‐dependently from the 10 and 20 μg groups, as compared to the LNP control, and no apparent further elevation was seen when the dose increased to 50 μg (saRNA‐50) (Figure 4c). Quantitative neutralization assays demonstrated progressive elevation of antibody titers through 35 dpv across all saRNA dose groups. While the titers were markedly lower with the 10 μg group, there were no statistical differences (*p* > 0.05) at all time points between the 20 μg (saRNA‐20) and the 50 μg (saRNA‐50) groups (Figure 4d). This plateau effect suggests potential optimization of the saRNA vaccine dosing regimen while maintaining immunogenicity.

To determine whether prime‐boost immunization was necessary, we compared single (sa‐Once) versus dual (sa‐Twice) vaccination (Figure 4e). The antibody responses in the sa‐Once and sa‐Twice groups were indistinguishable throughout the 28‐day observation period (Figure 4f), with both demonstrating superior responses versus CIV group at 21 and 28 dpv (*p* < 0.001). Similarly, there were no significant differences in IL‐4 and IFN‐γ concentrations between sa‐Once and sa‐Twice at 28 dpv, but significantly higher than the mock and CIV groups (Figure 4g). Pigs of the sa‐Once and sa‐Twice groups had similar neutralization antibody titers (*p* > 0.05), but the sa‐Twice group showed significantly higher titers than the CIV group at 28 dpv (*p* < 0.05) (Figure 4h). These findings support the feasibility of implementing a single vaccination schedule with saRNA vaccines while maintaining immunogenicity equivalent to dual vaccination.

Integrated analysis of the above trials demonstrated comparable humoral and cellular immune responses, between the group saRNA‐20 and group saRNA‐50 as well as between single and dual vaccinations at the 20 μg dose level. Therefore, a single 20 μg IM injection constitutes the optimal vaccination strategy that could provide equivalent immunogenicity.

### 3.6. PEDV‐S saRNA Vaccine Confers Protection Against PEDV Challenge in Piglets

**Figure 5:**
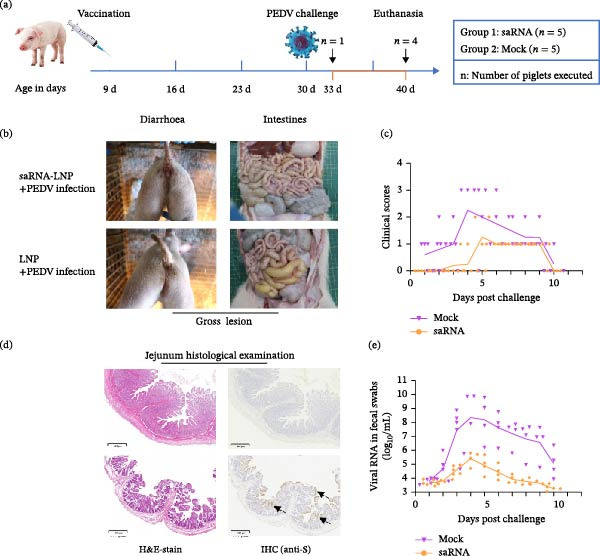
PEDV‐S saRNA vaccine confers protection against PEDV challenge in piglets. (a) Timeline of vaccination and challenge: piglets vaccinated at 9 days after birth, challenged with PEDV–WH at 21 dpv, and euthanized at 3 and 10 days post‐challenge (dpc). (b) Clinical observation and gross anatomy. (c) Clinical scores based on Table 1. (d) Jejunum thin sections H&E‐stained or specific antibody‐probed (IHC, immunohistochemistry). PEDV was shown as brownish‐gray color (arrows) at 3 dpc. (e) Viral shedding (*PEDV N* gene copies/mL suspensions of fecal swabs).

To assess the protective efficacy of the PEDV‐S saRNA vaccine against live virus challenge, piglets at 21 dpv were orally inoculated with the PEDV–WH strain and monitored for clinical signs, pathological lesions, and viral shedding (Figure 5a). Disease severity was evaluated using a scoring system based on mental state and diarrhea intensity (Table 1) [17, 23–25]. Vaccinated piglets displayed minimal clinical signs, with only one case of mushy feces (score = 1) observed during the first 3 days postchallenge (dpc), while no vaccinated animals exhibited diarrhea‐associated mental depression throughout the study period (Figure 5b,c). In contrast, control piglets demonstrated severe clinical manifestations, including watery diarrhea in three cases and lethargy in one case. Notably, all vaccinated piglets fully recovered by 10 dpc. To compare the gross and histological lesions in the immunized and control animals, one piglet, randomly selected from each group, was euthanized for necropsy at 3 dpc to reveal the distinctions between the two groups. No obvious gross lesions were observed in the intestinal tissues of the piglet from the saRNA group, while thin‐walled, gas‐distended intestines with minimal hemorrhage were apparent in the piglet from the control group (Figure 5b). The jejunum sections from the control piglet had blunted, fragmented and atrophied villi, as compared with absence of abnormal villi in the jejunum of saRNA‐immunized piglets. Immunohistochemistry revealed PEDV immunoreactivity in the cytoplasm of epithelial cells comprising the atrophied villi of the control piglet jejunum. By contrast, PEDV‐positive cells were scarcely detectable in the jejunal segments of the immunized piglet (Figure 5d). Analysis of PEDV RNA shedding revealed peak viral loads at 4 dpc in both groups, but the loads were substantially lower in vaccinated piglets than in controls (Figure 5e). These data suggest that the PEDV‐S saRNA vaccine reduces clinical signs and pathological lesions, and lowers viral load in actively immunized piglets following the PEDV challenge.

### 3.7. Humoral Immune Responses and Neutralizing Antibodies Against PED by saRNA Vaccination in Pregnant Sows

**Figure 6:**
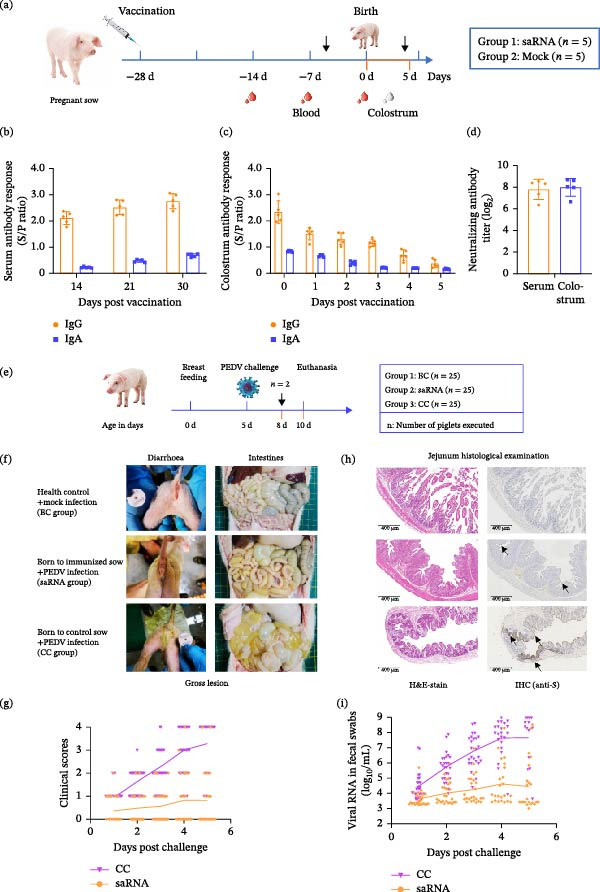
Immune response in sows inoculated with saRNA and its passive immune protective effect on suckling piglets via colostrum transfer. (a) Schedule: pregnant sows vaccinated 28 days prefarrowing (50 μg/pig). (b) Serum collected at −14, −7, and 0 days prefarrowing for PEDV‐S‐specific antibody responses. (c) Colostrum samples (Days 0–5) were assessed for PEDV‐S‐specific antibody responses. (d) Neutralizing antibodies against PEDV–WH strain in serum samples at farrowing and postpartum colostrum samples (Day 0). (e) Timeline: suckling piglets challenged with PEDV–WH strain at 5 days postpartum, and euthanized at Days 3 and 5 postchallenge (dpc). (f) Clinical observation and gross anatomy. (g) Clinical scores based on Table 1. (h) Jejunum thin sections H&E‐stained or specific antibody‐probed (IHC, immunohistochemistry). PEDV was shown as brownish‐gray color (arrows) at 3 dpc. (i) Viral shedding (PEDV *N* gene copies/mL suspensions of fecal swabs).

Having established the protective efficacy of the PEDV‐S saRNA vaccine in piglets, we next evaluated its immunogenicity in pregnant sows (Figure 6a). Five sows received IM vaccination (PEDV‐S saRNA, 50 μg/piglet), while five control sows received LNP. There were no adverse events observed postimmunization. Serological analyses showed that vaccinated sows exhibited a significant increase in PEDV‐S‐specific IgG and IgA antibody responses prior to farrowing, although serum IgA antibody levels did not reach the same height as IgG levels (Figure 6b). Analysis of lacteal secretions demonstrated that colostral IgG and IgA titers peaked at parturition and remained at high levels for the first 3 days postpartum (Figure 6c). Neutralization assay demonstrated potent activity against the PEDV–WH strain with a geometric mean titer of ~1:224 or 1:256 in serum and colostrum samples collected at farrowing (Figure 6d).

### 3.8. Passive Immunization of Suckling Piglets via Colostrum Transfer

We subsequently evaluated the passive protective efficacy of the PEDV‐S saRNA vaccine in lactating piglets through maternal immunization. Neonatal piglets from vaccinated and mock sows were challenged with the PEDV–WH strain at Day 5 postpartum (Figure 6e). At 2 dpc, only 40% of piglets in the immunized group exhibited mild diarrhea, whereas all piglets in the challenge control group displayed clinical symptoms including diarrhea, with some additionally presenting anorexia, vomiting, and tremors. About 80% of the challenge control group piglets died (beginning on the third dpc). To compare the histopathological lesions between the offspring of the immunized and the control sow, two piglets were randomly selected from each group—the immunized group, challenge control group, and blank control group—and euthanized for necropsy at 3 dpc. No obvious gross lesions were observed in the intestinal tissues of the piglets from the immunized sows. Thin‐walled and gas‐distended intestines were observed in the piglets from the challenge control sow (Figure 6f,g). Significant histopathological changes were observed in the jejunum of piglets in the challenge control group, including blunting of the villous architecture and atrophy while there were no obvious histopathological changes in the jejunum of the immunized group piglet (Figure 6h). Immunohistochemistry showed apparent PEDV distribution in the atrophied villi of jejunum from the challenge control piglet, while only a small fraction of PEDV‐positive cells was detected in the jejunum of the immunized group piglet. Compared with the challenge control group, viral loads in the fecal samples of piglets from the immunized group were significantly reduced (Figure 6i). The above findings demonstrate that passive immunization via colostrum from PEDV‐S saRNA‐vaccinated sows confers robust protection to suckling piglets against viral challenge, as evidenced by attenuated clinical symptoms, reduced viral shedding, and relatively intact intestinal morphology.

## 4. Discussion

PED causes severe economic losses in the global swine industry due to its high morbidity and mortality [34, 35]. Vaccination remains the primary strategy for PED prevention and control [18]. Given the rapid mutation rate of PEDV, nucleic acid vaccine offers a critical advantage due to their shorter development and production cycles, enabling prompt adaptation to emerging viral variants [15, 36, 37]. This adaptability is exemplified by the success of mRNA‐based COVID‐19 vaccines [38], suggesting that nucleic acid vaccine could become central to PED mitigation. Several mRNA vaccine candidates have demonstrated promise in preclinical studies. An LNP‐encapsulated mRNA vaccine encoding the S protein of the PEDV AH2012/12 strain could effectively stimulate both active and passive immunity in piglets [17]. Similarly, Yang et al. [39] designed an mRNA vaccine targeting the receptor‐binding domain (RBD) of the PEDV NB‐F71 strain and found that dimeric RBD mRNA combined with a live‐attenuated vaccine‐elicited antibody titer comparable to an inactivated vaccine. However, nonreplicating mRNA vaccine exhibits limitations [40], including transient antigen expression, as demonstrated by in vivo murine imaging studies (Figure 1b,c). saRNA vaccine addresses this constraint by sustaining intracellular antigen production, thereby prolonging immunogenic exposure [41, 42]. Among several saRNA constructs evaluated in this study, saRNA‐2 group displayed delayed but prolonged‐expression kinetics. While initial fluorescence levels were moderate, it uniquely persisted until Day 8 and exhibited the latest signal decay (Figure 1). Based on these findings, we selected saRNA‐2 for further platform‐derived PEDV vaccine development.

The full‐length S protein of PEDV is composed of S1 and S2 that contain all the antigen epitopes. S1 region harbors multiple neutralizing epitopes [43, 44], and immunodominant neutralizing epitopes have been identified in the N‐terminal portion of S2 domain [45, 46]. Due to these characteristics, both S1 and S2 domains have been prioritized in recent platform‐derived PEDV vaccine development efforts. While previous studies have demonstrated that full‐length S mRNA vaccines elicit higher neutralizing antibody titers compared to truncated versions [17], our investigation revealed an important exception in the context of saRNA vaccines, that is, the truncated S(1–785aa) saRNA vaccine candidate elicited higher antibody responses and neutralizing antibody titers than its full‐length counterpart (Figure 3b,c), suggesting that immunogenicity of the target antigen fragments might differ with different vaccine platforms that should be taken into account for saRNA‐based vaccine development. The extended length of saRNA (approximately fivefold longer than nonreplicating mRNA) increases its susceptibility to nuclease degradation, while the introduction of replicase sequences results in the formation of complex secondary structures, which may affect RNA stability [47–49]. Therefore, this difference may stem from reduced expression efficiency of the full‐length S saRNA due to excessive exogenous sequence length which could impair RNA stability or translational capacity. Nevertheless, these limitations can be partially counterbalanced through molecular optimization strategies [50, 51]. Furthermore, this experiment demonstrated that specific antibody responses in piglets immunized with the truncated S saRNA vaccine remained high at 70 dpv (Figure 3b), confirming sustained expression of the antigen, a key advantage of protective immunity.

The saRNA vaccine platform inherently elicits robust and sustained immune responses at significantly lower doses compared to conventional vaccines [41, 52, 53]. In this study, we optimized the immunization protocol for our PEDV‐S targeting saRNA vaccine. We have demonstrated that a single low‐dose administration effectively induces robust active and passive immunity in piglets, conferring protection upon viral challenge. Notably, this contrasts with commercially available PEDV vaccines, which predominantly require prime‐boost regimens to achieve comparable efficacy [54, 55]. By eliminating the need for multiple vaccinations, our saRNA‐based approach not only streamlines immunization times but also reduces production costs through lower antigen and adjuvant requirements.

The SARS‐CoV‐2 saRNA vaccine candidate elicited robust antibody and cellular immune responses and induced both Th1 and Th2 IgGs, though Th1‐biased [7]. Our results demonstrated that the PEDV‐S saRNA vaccine generated more neutralizing antibody potency and specific IgG antibodies comparing to CIVs. Unfortunately, the specific IgA antibodies induced by PEDV‐S saRNA vaccine in pregnant sows were not as high as those of IgG, which may be attributed to the fact that we employed an IM immunization route, not mucosal immunity [56, 57]. The vaccine presented efficient protection against PEDV challenge, suggesting that protective efficacy may primarily be due to a synergistic combination of neutralizing antibodies and cellular immune responses [17]. Specifically, we observed significant activation of both CD4^+^ and CD8^+^ T cells, accompanied by elevated serum levels of IL‐4 and IFN‐γ in immunized piglets. Flow cytometry analysis revealed a modest Th1‐biased response, though this Th1 polarization was less pronounced than anticipated (Figures 3g,h). Unfortunately, the present study did not comprehensively investigate the cellular immune mechanisms underlying saRNA vaccine‐mediated protection, warranting further detailed exploration in future study.

Passive immunization plays a critical role in protecting neonatal piglets against PEDV infection [21, 44, 58, 59]. Our study demonstrated that immunization of sows with the PEDV‐S saRNA vaccine effectively induced PEDV‐specific IgG and IgA in both serum and colostrum. Although the levels of these specific antibodies are relatively lower than those in other inactivated vaccine studies, lactating piglets born to vaccinated sows exhibited 88% survival rate against PEDV challenge, accompanied by a significant reduction in fecal PEDV RNA levels [20]. These findings suggest that the vaccine elicits a robust neutralizing antibody response in sows, which in turn is efficiently transferred to piglets via colostrum, conferring protective immunity against PEDV infection.

## 5. Conclusions

In summary, this study highlights the superior efficacy of the PEDV‐S saRNA vaccine over commercial inactivated and nonreplicating mRNA vaccines. Furthermore, the results underscore the potential of saRNA technology as a promising platform for cost‐effective veterinary vaccine development.

## Author Contributions

Jinqi Shu, Chao Wu, and Xiaomin Zhao conceived and designed the experiments. Jinqi Shu, Fang Wang, Hanyu Zhang, Xin Liu, Keshu Liu, Yinhe Zha, Wenqin Chai, Jianghui Yao, and Yujun Lin performed the experiments. Jinqi Shu, Fang Wang, Hanyu Zhang, Chao Wu, and Xiaomin Zhao analyzed the data. Jinqi Shu, Han Zhang, and Xiaomin Zhao wrote the manuscript.

## Acknowledgments

The authors have nothing to report.

## Funding

This study was supported by the Shaanxi Province Innovation Capacity Support Program (Grant:2025JC‐GXPT‐018), Joint Development of Biological Products for Animals (Grant: K4050722009), and Special Fund Project for Animal Epidemic Prevention of Shaanxi Province (Grant: XNDY‐DF2401).

## Ethics Statement

Animal experiments were conducted by protocols approved by the Institutional Animal Care and Use Committee (IACUC) of Hangzhou ISEEVAX Pharmaceutical Technology Co., Ltd (Approval Number. ISEEVAX‐MO‐2024‐015 and ISEEVAX‐SW‐2024‐011). Strict measures were implemented to ensure animal welfare, including the minimization of animal numbers.

## Conflicts of Interest

The authors declare no conflicts of interest.

## Supporting Information

Additional supporting information can be found online in the Supporting Information section.

## Supporting information


**Supporting Information** Figure S1: Microscopic pictures for PEDV CPE in Vero cells at 100× magnification power. (a) Uninfected Vero cells. (b) Infected cells showing swelling and syncytium formation 48 h postinfection.

## Data Availability

The data that support the findings of this study are available from the corresponding author upon reasonable request.
