# Supplementary material for: A Self‐Amplifying RNA Lipid Nanoparticle (saRNA‐LNP) Vaccine Provides Effective Protection Against Porcine Epidemic Diarrhea
Source: Transbound Emerg Dis. 2026 May 6;2026:3115893. doi: 10.1155/tbed/3115893 (PMC13147209; doi:10.1155/tbed/3115893)
Supplement: Supplementary file 1 — Supporting Information Figure S1: Microscopic pictures for PEDV CPE in Vero cells at 100× magnification power. (a) Uninfected Vero cells. (b) Infected cells showing swelling and syncytium formation 48 h postinfection. [file TBED-2026-3115893-s001.docx]

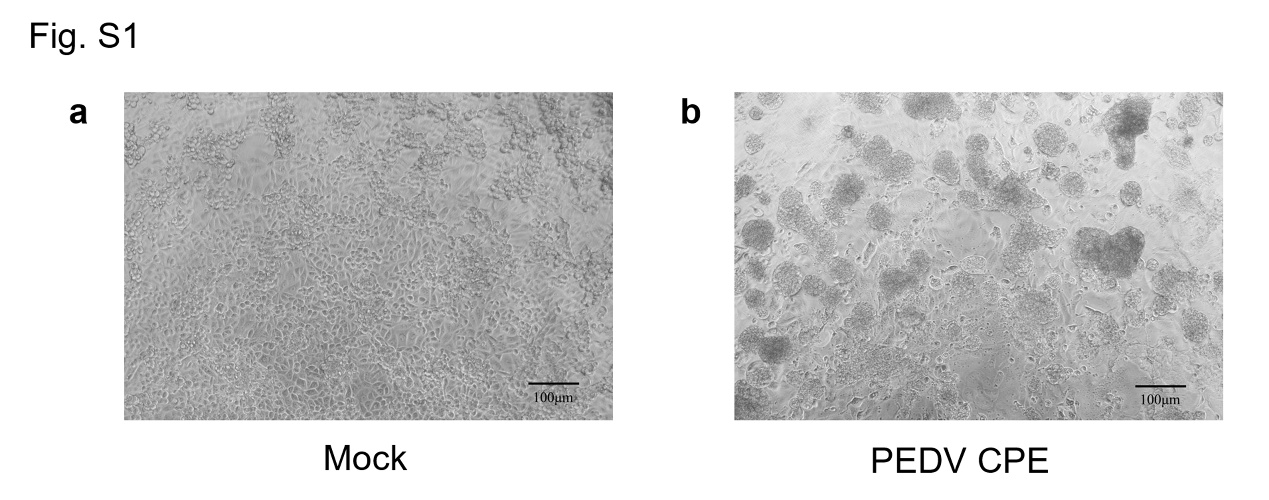
**Fig. S1. Microscopic pictures for PEDV CPE in Vero cells at 100× magnification power. (a) Uninfected Vero cells. (b) Infected cells showing swelling and syncytium formation 48 h post-infection.**
